# Analyzing histone ChIP-seq data with a bin-based probability of being signal

**DOI:** 10.1371/journal.pcbi.1011568

**Published:** 2023-10-20

**Authors:** Vivian Hecht, Kevin Dong, Sreshtaa Rajesh, Polina Shpilker, Siddarth Wekhande, Noam Shoresh

**Affiliations:** Gene Regulation Observatory, Broad Institute of MIT and Harvard, Cambridge, Massachusetts, United States of America; Academy of Mathematics and Systems Science, Chinese Academy of Science, CHINA

## Abstract

Histone ChIP-seq is one of the primary methods for charting the cellular epigenomic landscape, the components of which play a critical regulatory role in gene expression. Analyzing the activity of regulatory elements across datasets and cell types can be challenging due to shifting peak positions and normalization artifacts resulting from, for example, differing read depths, ChIP efficiencies, and target sizes. Moreover, broad regions of enrichment seen in repressive histone marks often evade detection by commonly used peak callers. Here, we present a simple and versatile method for identifying enriched regions in ChIP-seq data that relies on estimating a gamma distribution fit to non-overlapping 5kB genomic bins to establish a global background. We use this distribution to assign a probability of being signal (PBS) between zero and one to each 5 kB bin. This approach, while lower in resolution than typical peak-calling methods, provides a straightforward way to identify enriched regions and compare enrichments among multiple datasets, by transforming the data to values that are universally normalized and can be readily visualized and integrated with downstream analysis methods. We demonstrate applications of PBS for both broad and narrow histone marks, and provide several illustrations of biological insights which can be gleaned by integrating PBS scores with downstream data types.

## Author summary

Histone modifications are key to epigenetic regulation of gene expression. Their genomic distributions are most commonly measured using ChIP-seq, a next-generation sequencing method which results in pileups of sequencing reads near regions with modified histones. Comparing the signal between histone modification profiles in different cellular contexts is often challenging due to the lack of a clear standard for interpreting signal strength, the inherent variability in the positions of histones, and the difficulty of detecting very broad regions that are only modestly enriched compared to the background. We present a method, which we call PBS, or probability of being signal, to simplify and streamline the process of identifying where true signal can be found in ChIP-seq datasets, regardless of whether the signal is broad or narrow. We demonstrate how our method can be used to visualize, quantify and compare signal among ChIP-seq datasets, and to easily integrate ChIP-seq data with additional data types. We show how PBS can both stand on its own, and also add power to other commonly used analysis methods. We anticipate that its versatility and straightforward interpretation will prove useful in many applications.

## Introduction

Chromatin immunoprecipitation with sequencing, or ChIP-seq, is the one of the primary methods for mapping the chromatin landscape, a key element of gene-regulatory circuitry [1]. ChIP-seq is frequently used to interrogate chemical modifications on the tails of histone structural proteins, which play a central role in determining the function of regulatory elements. Analyzing ChIP-seq data typically starts with identifying regions of enriched signal via peak calling or segmentation, and often continues with comparing the signal among multiple cellular contexts or integration with other data types [2, 3]. While a variety of tools to accomplish these objectives already exist, they do not fully address the particular analytical challenges posed by histone modification (or histone mark) ChIP-seq.

Most approaches for quantitatively identifying regions with ChIP-seq enrichment for a particular histone mark involve calling peaks. However, peak callers, initially developed for transcription factor ChIP-seq, can be limited in certain contexts [4, 5]. In particular, histone modifications with very broad, flat regions of enrichment may remain undetected by many peak callers, which typically search for defined regions with elevated signal compared to a local background [6]. Fine-tuning parameters or using specialized peak callers can partially improve detection of broad marks, but implementing such changes over large numbers of datasets and in analysis pipelines quickly becomes cumbersome.

Using peak calling as a basis for determining enrichment also complicates comparing enriched regions across multiple histone ChIP-seq datasets. Unlike transcription factors, whose binding site is at a specific genomic location, nucleosome positions are not similarly fixed and can vary across cells and samples. Thus, a signal shared across multiple datasets may appear to split into multiple signals or shift position, even in the absence of a true difference.

A variety of tools exist to quantitatively compare ChIP-seq tracks, many of them adaptations of methods developed for detecting differential gene expression [7–12]. These methods generally take lists of peaks as input, and return those that are significantly differentially enriched; although many methods use similar underlying algorithms, most do not produce concordant results when provided identical data [7].

Finally, integrating ChIP-seq data with additional datasets, such as other epigenetic data types or variants identified in genome-wide association studies (GWAS), provides genomic context essential for mechanistic insight [13]. Currently, most examples of these integrative analyses involve considerable parameter tuning, particularly regarding how to define an overlap or colocalization between two or more features. A convenient, straightforward, and consistent way to consolidate ChIP-seq with these heterogeneous data sources could meaningfully facilitate downstream analyses.

Here, we present a bin-based method for analyzing ChIP-seq data that can identify regions of enrichment in a wide range of histone marks. We split the genome into non-overlapping 5 kB bins, and for each 5 kB bin we calculate a probability of being signal (PBS), based on an estimate of a genome-wide background. We demonstrate that PBS can be used to detect regions of low, broad signal that is characteristic, for example, of H3K27me3 signal, as well as more punctate signal regions, such as those observed in narrow histone marks including H3K27ac. We show how combining PBS values with called peaks improves the interpretability of peak-based differential enrichment analysis. Finally, we combine PBS with GWAS SNPs, illustrating how PBS empowers rapid and straightforward integrative analyses.

## Results

### Method overview

**Fig 1:**
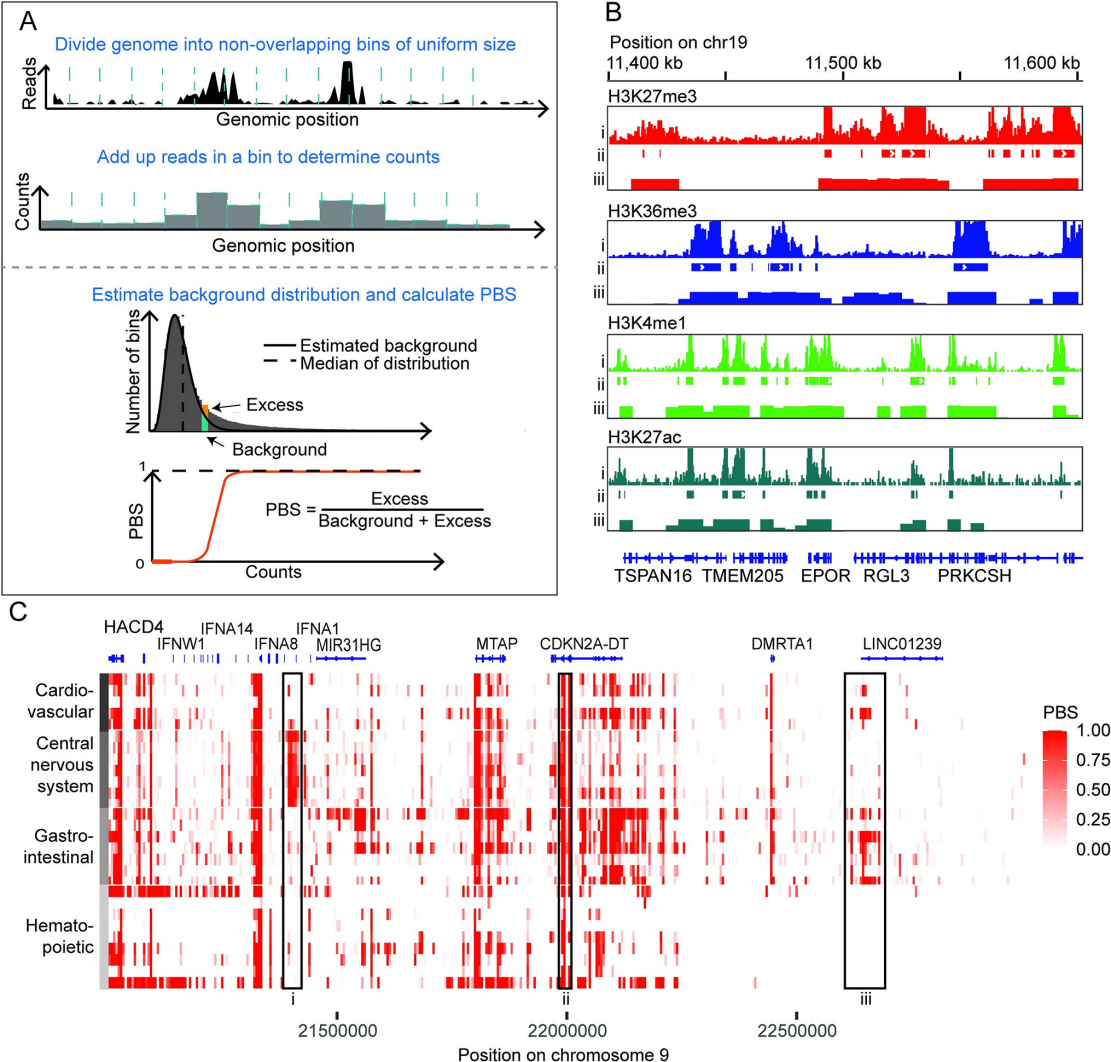
Using PBS to identify signal enrichment in ChIP-seq datasets. (A) Schematic of the PBS method. (B) Signal tracks (i), called peaks (ii) and PBS values (iii) for several histone modifications at a sample locus in tissue from esophagus. This example was chosen for demonstration as the locus has signal from both narrow and broad histone marks in this tissue. (C) Heatmap of a collection of 28 H3K27ac datasets from four different tissue categories showing signal at the CDKN2A locus. Highlighted boxes demonstrate enrichment of H3K27ac signal proximal to IFNA8 in central nervous system tissues (i), CDKN2A in all tissue categories (ii), and LINC01239 in gastrointestinal and cardiovascular tissues (iii).

To calculate PBS, the genome is first divided into non-overlapping 5 kB bins (Fig 1A). This bin size is appropriate for capturing signal from most broad and narrow ChIP-Seq peaks, without any additional parameter tuning (Fig 1B). This absence of parameter tuning makes PBS particularly simple and straightforward to implement, even in pre-existing bioinformatics pipelines. Binning the data acts as a low-pass filter, bypassing issues associated with inconsistencies in nucleosome positioning, and resulting in a less precise but more robust representation of the data when compared with peak calling. Smaller or larger bin sizes can be used, though decreasing the bin size below 3 kB may require some adaptation of the method. The number of reads overlapping each bin, or the read-counts, is calculated, then rescaled by the average mappability and copy number of each bin. Bins with low mappability, arising from a non-unique underlying sequence, will often have an underestimate of the total number of reads, since many reads that would otherwise be align to them are filtered out. Additionally, copy number variations, often observed in tumor samples, may artifactually increase or decrease the amount of signal due to changes in genomic content (S1 Fig). Correcting for these effects improves the estimate of the signal in each bin. These rescaling steps are described in greater detail in the Methods section.

**Fig 2:**
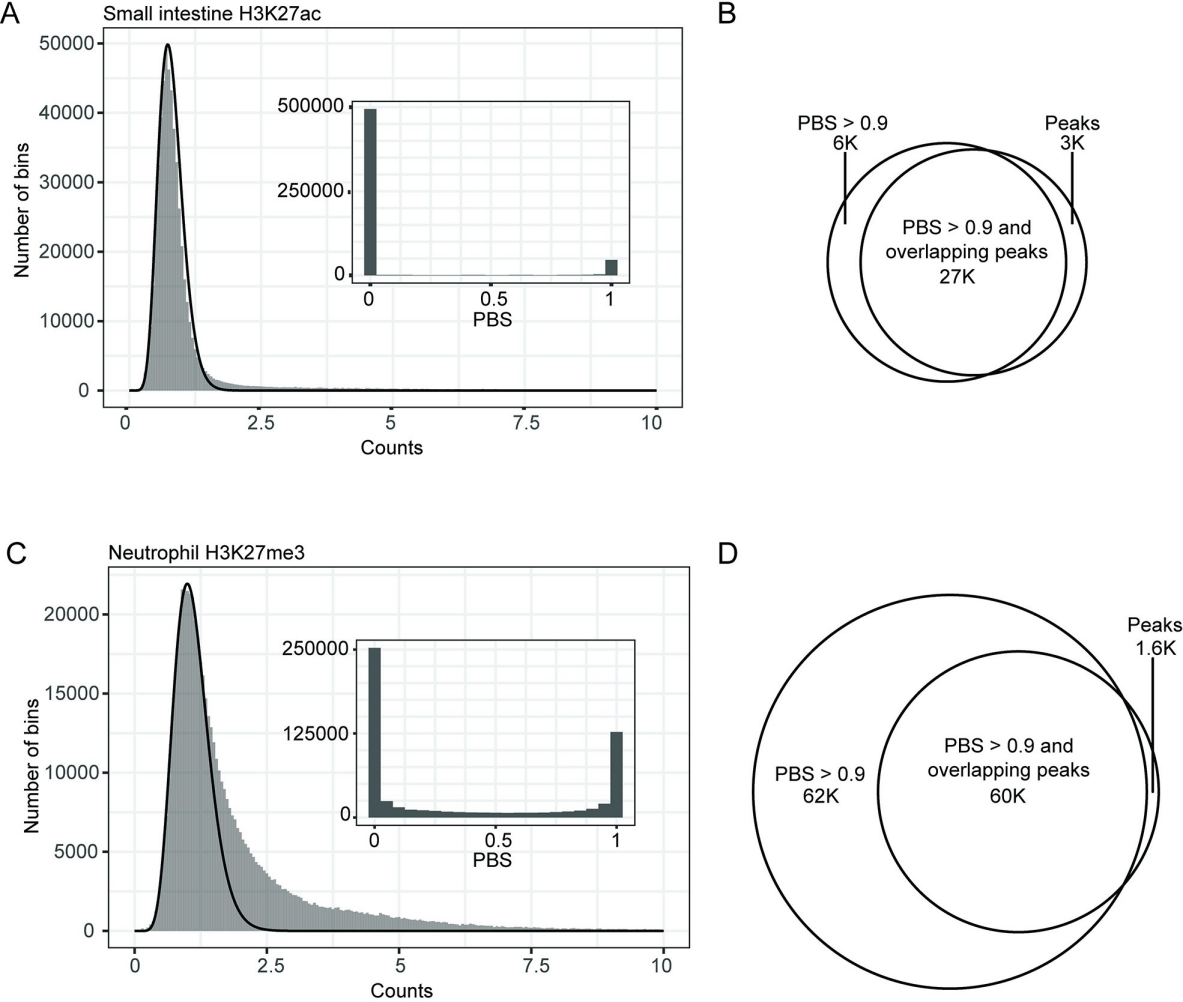
Comparing PBS and peak calling in H3K27ac and H3K27me3 datasets. (A) Histogram of read-counts and PBS (inset) for a representative H3K27ac dataset. (B) Venn diagram describing overlap between bins with high PBS and bins with peaks in (A). (C) Histogram of read-counts and PBS (inset) for a representative H3K27me3 dataset. (D) Venn diagram describing overlap between bins with high PBS and bins in (C). In both (A) and (C), the black curve corresponds to the background distribution estimated from a fit to bottom fiftieth percentile of data. The heavier tail and greater number of bins with PBS > 0 in (C) compared to (A) indicate elevated genome-wide enrichment of H3K27me3 when compared to H3K27ac. Venn diagrams to the right of the histograms indicate the overlap between bins containing peaks and those with PBS > 0.9 for each histone mark.

Once the read-counts have been rescaled, the background distribution can be estimated as follows. The distribution of read-counts is positively skewed, with a long right-hand tail (Fig 1A, bottom panel). It is made up of two components: the background distribution comprised of all the bins that do not actually contain the ChIP target, and the remaining, signal, distribution. As the characteristics of the signal portion of the distribution vary considerably among experiments, ChIP targets and antibodies, we attempt to parametrize only the background part of the distribution. While these two components of the distribution do typically overlap somewhat, for any reasonable ChIP, the bins with low counts, making up the left-hand side of the distribution, would belong mostly, if not entirely, to the background distribution. Using this fact, we estimate the background portion by fitting a gamma distribution to the bottom fiftieth percentile of the data (Fig 2A and 2B and Methods). It should be noted that the choice of fiftieth percentile is heuristic and represents a tradeoff between trying to include as much data as possible to improve the parameter estimation while still avoiding signal bins, and it is possible to adjust this cutoff in cases where it is deemed useful. We can confirm that fitting to the bottom half of the data is adequate to estimate the background by inspecting the fit to an input control, which is entirely background and fits the estimated gamma distribution almost perfectly (S2 Fig). PBS is calculated using this distribution, along with the empirical distribution of all values of read-counts, as the difference between the values of the empirical and estimated distributions divided by the empirical distribution at a given value of read-counts. Thus, for the low read-counts background bins, the empirical and estimated distributions are equal to each other, and PBS is zero. For counts corresponding to enriched regions, the resulting PBS value is the fraction of excess bins at that signal level that cannot be explained by the background distribution.

**Fig 3:**
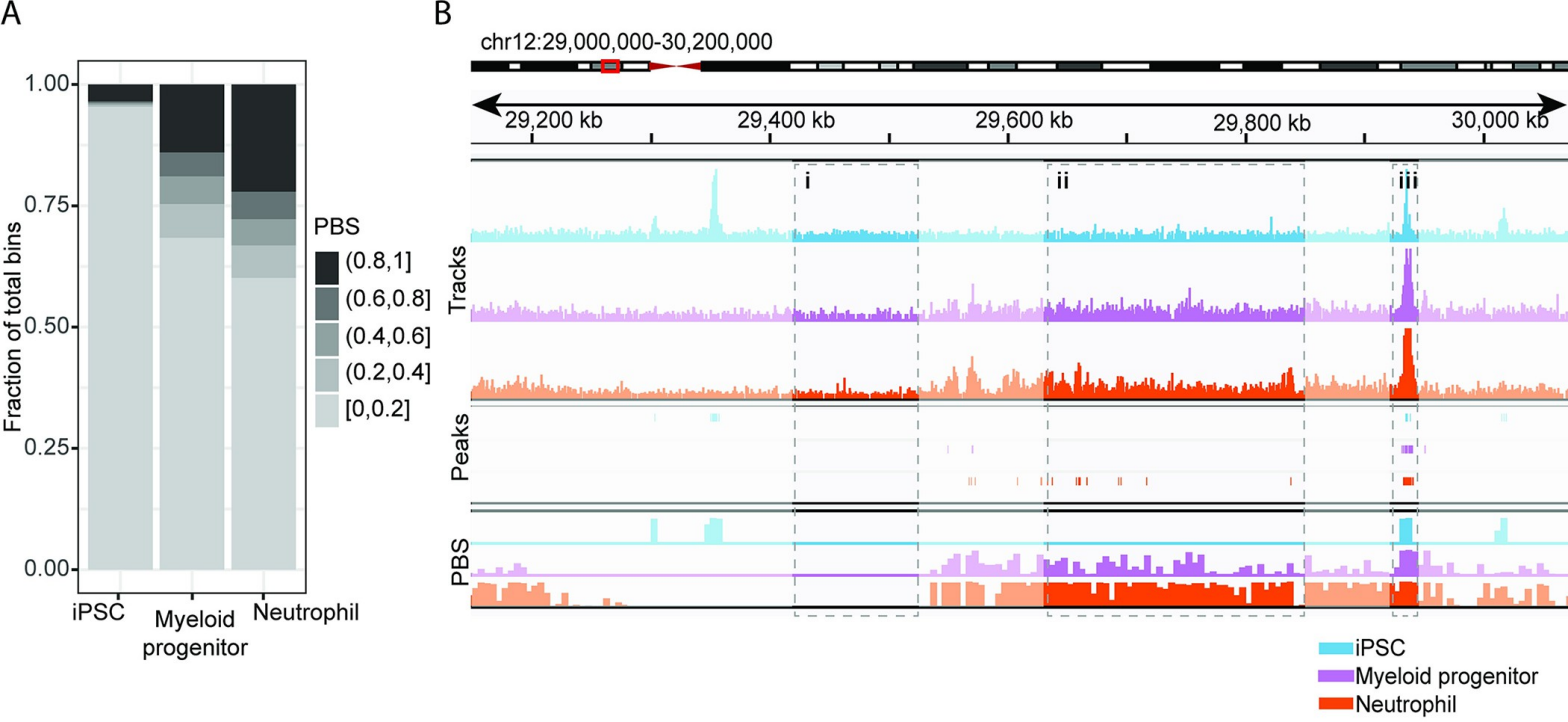
Using PBS to detect changes in H3K27me3 levels during cell differentiation. (A) Summary of genome-wide PBS across H3K27me3 datasets of iPSCs, myeloid progenitors, or neutrophils. The number of bins with high PBS (> 0.8) increases with increased cell differentiation. Additionally, moderate levels of PBS (between 0.2 and 0.8) corresponding to low, broad H3K27me3 signal, appear exclusively in more differentiated cell types (myeloid progenitor cells and neutrophils). (B) Comparing iPSC, myeloid progenitor cells and neutrophils, including signal tracks (top), MACS2 peaks (middle), and PBS (bottom). Highlighted are regions with no signal in all three datasets (i); no signal in iPSC but moderate signal in myeloid progenitors and moderate-high signal in neutrophils (ii); and high signal in all three cell types (iii). While both peak calling and PBS identify the consistent sharper peak in (iii), only PBS meaningfully detects the spreading enrichment in (ii) (14 peaks, spanning 3.8% of the region vs. 74% of the region with PBS equal to 0.8 or higher).

**Fig 4:**
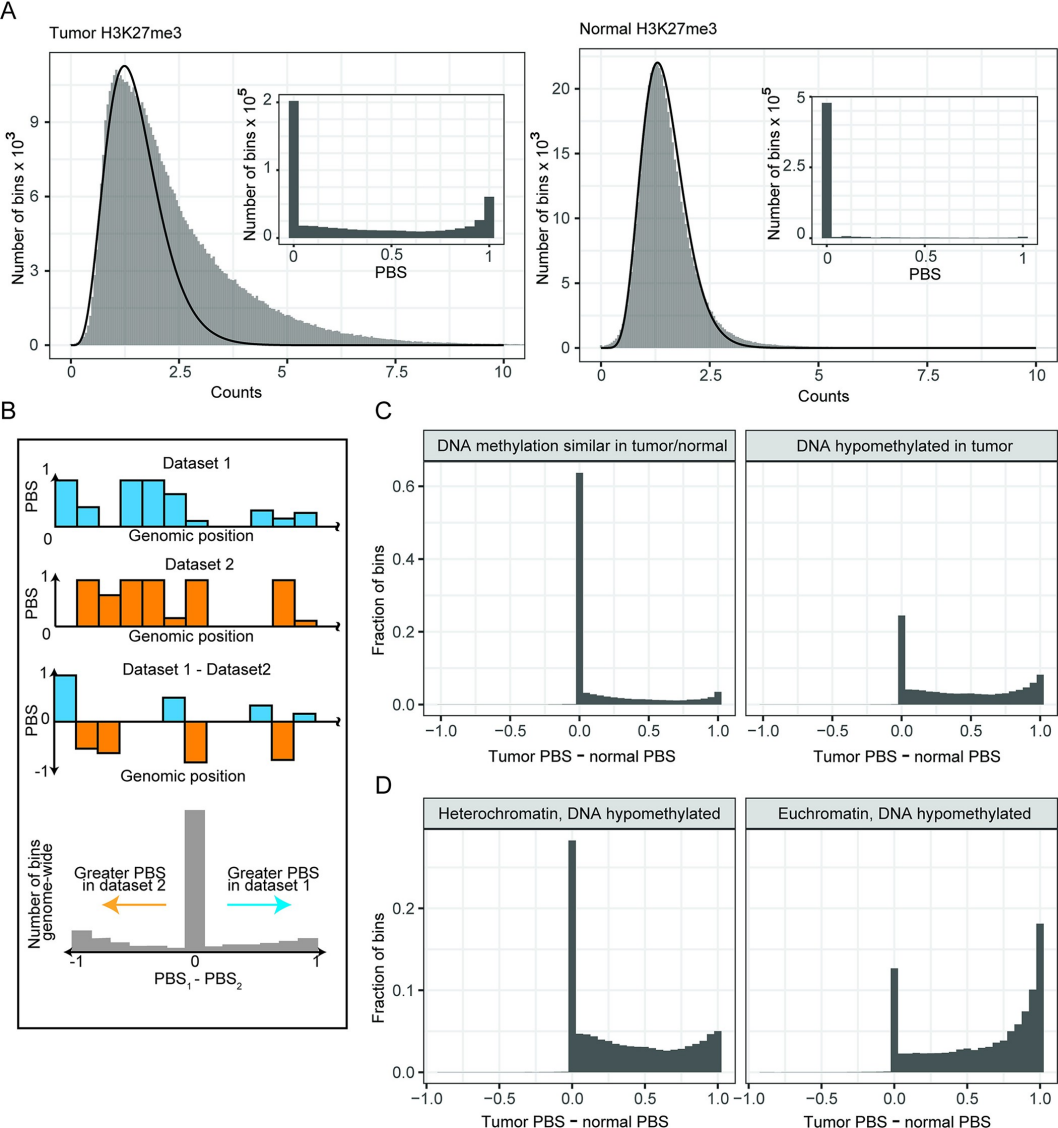
Detecting elevated H3K27me3 in colon tumor samples. (A) Comparing distributions of counts for H3K27me3 in tumor (left) and normal (right) colon tissue. Tumor tissue appears to have globally elevated H3K27me3, as shown by the heavier tail in the histogram of counts, corresponding to a greater number of non-zero PBS values (inset). (B) Schematic describing a PBS-based differential analysis of two datasets. The output of the differential analysis is a histogram of the differences in PBS between the two datasets, as shown at the bottom. (C) Histograms summarizing a PBS-based differential analysis of H3K27me3 PBS between tumor and normal colon tissue. Plots are facetted based on overlap with a region of hypomethylated DNA (hypomethylated block). (D) Histograms describing a PBS-based differential analysis of H3K27me3 between tumor and normal colon tissue in hypomethylated blocks (right-hand facet in (C)). Plots are facetted based on overlap with euchromatin or heterochromatin, as defined by Hi-C. The increase in H3K27me3 in tumor tissue is more pronounced in regions of euchromatin that overlap hypomethylated tumor DNA. Hypomethylated tumor DNA is defined as a 5 kB bin with methylation levels lower than those of normal tissue [24].

The PBS calculation can be interpreted directly as the probability that a bin containing a certain number of reads has a true signal of enrichment. Bins with PBS of zero likely have no signal, bins with PBS equal to one almost certainly contain signal (Fig 2A and 2B). Signal from narrower histone marks such as H3K27ac or from ATAC-seq tends to follow a nearly bimodal distribution (Figs 2A and S3), with most PBS values concentrated either close to zero or close to one. Broader histone marks such as H3K27me3 often have more genomic regions with intermediate values of PBS (Figs 2C, 3A and 4A). When clearly bimodal, it is often reasonable to set a threshold and to treat PBS as a binary, on/off signal. In other cases, and depending on the question being addressed, and the character of the histone mark, it may be more appropriate to use the full range of PBS values. In the rest of this manuscript, we provide some guiding examples.

In addition to quantitative applications, we also find PBS to be very useful for qualitatively inspecting the chromatin landscape of large genomic regions, for example by converting the PBS values into a heatmap, such as is shown in Fig 1C. This format is compact and also does not require additional normalization of the data, thereby simplifying the interpretation of the visualization. In the example shown in Fig 1C, we use continuous values of PBS to examine a gene-rich 2 MB region in chromosome 9 surrounding the CDKN2A locus across 28 different tissue types in four different tissue categories (cardiovascular, central nervous system, gastrointestinal, and hematopoietic), and identify regions with both tissue-specific and non-specific H3K27ac enrichment. We can relate these enrichments to previously identified roles of these genes. For example, CDKN2A is a cell cycle regulatory protein implicated in a wide range of cellular behaviors and diseases impacting many different tissue types [14–16]. Conversely, IFNA8 shows selective H3K27ac in central nervous system tissue types [17], while LINC01239 shows enrichment in gastrointestinal and cardiovascular tissue types [18], a pattern consistent with the roles of these genes as described in literature and as seen in gene expression described in GTEx [19]. IFNA8 encodes a cytokine responsible for lymphocyte activation in response to infection, and has been shown to play a role in glioma, a cancer of the central nervous system, with mutations in its promoter region affecting patient survival [17]. Additionally, LINC01239 has been reported as linked to colorectal adenomas and certain types of liver cancers [20, 21]. Further information regarding its biological function remains limited.

### Comparing PBS with MACS2 peak calling

As PBS is a method for identifying signal-enriched regions in the genome, we compared it to peak calling, a more common method for accomplishing this purpose. Specifically, we compared PBS values with peaks called by MACS2 for several tissue types and histone modifications [5]. We annotated each 5 kB bin by whether or not it overlapped with a peak detected by MACS2, and visually inspected the fit of the estimated background distribution to the distribution of bins that did not contain any peaks (S4 Fig). In examining a representative distribution of H3K27ac, we found that most bins had PBS of either 0 or 1 (Fig 2A), consistent with our expectation of the punctate nature of a narrow histone mark. Bins with low PBS (between 0 and 0.1) tended to rarely overlap with peaks called by MACS2 (0.2% of bins), whereas close to 90% of bins with PBS between 0.9 and 1 overlapped with peaks, suggesting good agreement between the two methods (Figs 2B and S5). We expected to find peaks primarily in bins with PBS > 0.9, as these have the highest probability of including signal (S5 Fig).

However, when evaluating broader histone marks, such as H3K27me3, we noticed that certain datasets had a considerably larger number of bins with PBS > 0.9 not overlapping with peaks (Fig 2C and 2D). We observed this difference in spite of using the “—broad” flag for MACS2, the recommended setting for broad peaks [5]. Additionally, we observed a large number of bins with PBS between 0.1 and 0.9, as indicated by the inset histogram in 2C (approximately 24% of all bins). We hypothesized that these bins would correspond to repressed broad regions of low levels of H3K27me3 found in terminally differentiated cell and tissue types [22, 23]. Many peak callers rely on measuring deviations from background in a local window which is usually considerably smaller (<10 kb) than the size of the broadly enriched regions (> 1 MB). Thus, these low, flat regions are particularly difficult for many peak callers to detect. Because PBS values are determined relative to a genome-wide background, even low levels of enrichment spanning broad regions can still be detected.

### Detecting H3K27me3 ChIP-seq signal with PBS

To examine further the characterization of broad, low-enrichment regions, we considered a set of three cell types—iPSCs, myeloid progenitors, and neutrophils—representing levels of differentiation starting from multipotent and ending at terminally differentiated. To simplify the comparison between datasets while still enabling us to examine the moderate levels of PBS with high enough resolution, we binned the PBS values into five groups, each with a span of 0.2 units (Fig 3A). We found that the iPSCs had the least widespread high H3K27me3 signal genome-wide as measured by PBS > 0.8, followed by the myeloid progenitors and finally the neutrophils (Fig 3A). Moreover, unlike the myeloid progenitors and neutrophils, the iPSCs also had few regions with moderate levels of H3K27me3, as indicated by PBS between 0.2 and 0.8. We confirmed this pattern by visually inspecting the tracks (Fig 3B). We found considerable evidence of moderate H3K27me3 enrichment in the neutrophils (PBS between 0.2 and 0.8 in 17.8% of genome-wide bins). This moderate enrichment was present to a similar extent (corresponding to 17.6% of genome-wide bins) in the myeloid progenitors, and nearly absent in the iPSCs (1.1% of total bins); though this signal was reflected by elevated PBS, it remained generally undetected by peak calling (Fig 3B).

Elevated genome-wide levels of H3K27me3 have been observed in other contexts, including recently in colorectal carcinomas [24]. We sought to recapitulate the analysis described in [24] with PBS, in the process demonstrating how PBS can be used to simply and easily integrate disparate data types. When visually inspecting the signal tracks on a genome browser, we observed low, broad H3K27me3 enrichment in tumor samples compared to normal. We were then able to substantiate this observation by examining the heavy right-hand tail in the tumor read-counts distribution, as well as the considerably higher number of non-zero PBS bins compared to the normal sample (Fig 4A). To identify regions of differential H3K27me3 enrichment, we performed a differential PBS analysis, which involves simply subtracting two PBS datasets on a bin-by-bin basis (Fig 4B). This approach provides a near-instant list of 5 kB regions that are enriched for signal in one sample or another. Genomic annotations in tabular format, such as those that can indicate whether differential signal is concentrated in particular genomic contexts, can be integrated in a straightforward manner. Here, we show an example of how the PBS differential analysis can be conducted with continuous values of PBS, due to the relatively large number of bins with moderate levels of PBS (Fig 4A). However, the subtracted PBS values can also be thresholded with a similar intuition to previously discussed thresholds in PBS: regions with signal in one dataset and not the other will have a difference in PBS of magnitude close to 1.

We leveraged this workflow to investigate the differential H3K27me3 enrichment between tumor and normal samples, and how this differential enrichment might change in regions depleted of DNA methylation in the tumor samples. These hypomethylated blocks have been defined as having methylation differences larger than 10% between tumors and normal samples [24]. Johnstone et al in [24] hypothesized that elevated H3K27me3 could serve as an alternative means of repressing gene expression when DNA methylation is depleted, as frequently occurs in various tumor types [25]. Through the differential PBS analysis of tumor and normal samples, we found elevated H3K27me3 in tumor relative to normal genome-wide, though with a higher proportion of bins with PBS > 0.9 in hypomethylated blocks relative to non-block regions (17% within the hypomethylated blocks vs 6% outside the blocks, p < 2 x 10^−16^, Fisher’s exact test) (Fig 4C). When we looked more closely at the hypomethylated blocks, we found that the increase in proportion of genomic bins with elevated H3K27me3 in tumor relative to normal was more pronounced in blocks overlapping with euchromatin, or chromatin enriched for genes and often available for gene expression, relative to heterochromatin (32% vs 12%, p < 2 x 10^−16^, Fisher’s exact test) (Fig 4D). This observation, as described in [24], was particularly surprising considering that H3K27me3 is most often found in heterochromatin, and is generally absent from euchromatin, thereby supporting the notion of H3K27me3 serving as a substitute repressor of gene expression following depletion of DNA methylation in a tumor context.

### Synergy between PBS and peak-based differential enrichment methods

Quantitatively identifying regions of differential enrichment between pairs of ChIP-seq datasets is critical for understanding a broad range of biological effects and conditions. DiffBind, an R package commonly used for this type of analysis, relies on comparisons between lists of peaks, which are input into DESeq2 to identify regions of differential enrichment [7, 9, 26]. Though DiffBind has been shown via simulations to have good power for detecting differentially enriched peaks in narrow histone marks, the total number of significantly differentially bound regions can be very large, even after adjusting for multiple hypotheses [27]. As a result, those interpreting the results often set an arbitrary threshold to limit the number of regions of interest for downstream analysis, either based on the several dozen highest ranking regions, or with a heuristic for maximum FDR-adjusted p value.

We examined an alternative approach to focusing the results from DiffBind by filtering the DiffBind output with a PBS-based differential analysis (Fig 4B). A PBS-based differential analysis identifies bins that have signal in one sample and not in another sample; DiffBind, by contrast, typically includes regions that have different magnitudes of enrichment as significant. For histone modifications, a difference in peak magnitude alone is not always the best indicator of a difference in chromatin state [28]. Thus, by focusing on regions identified as differentially enriched by both PBS and DiffBind, we can limit the differential regions to ones that show a clear “on-to-off” or “off-to-on” transition between the samples.

**Fig 5:**
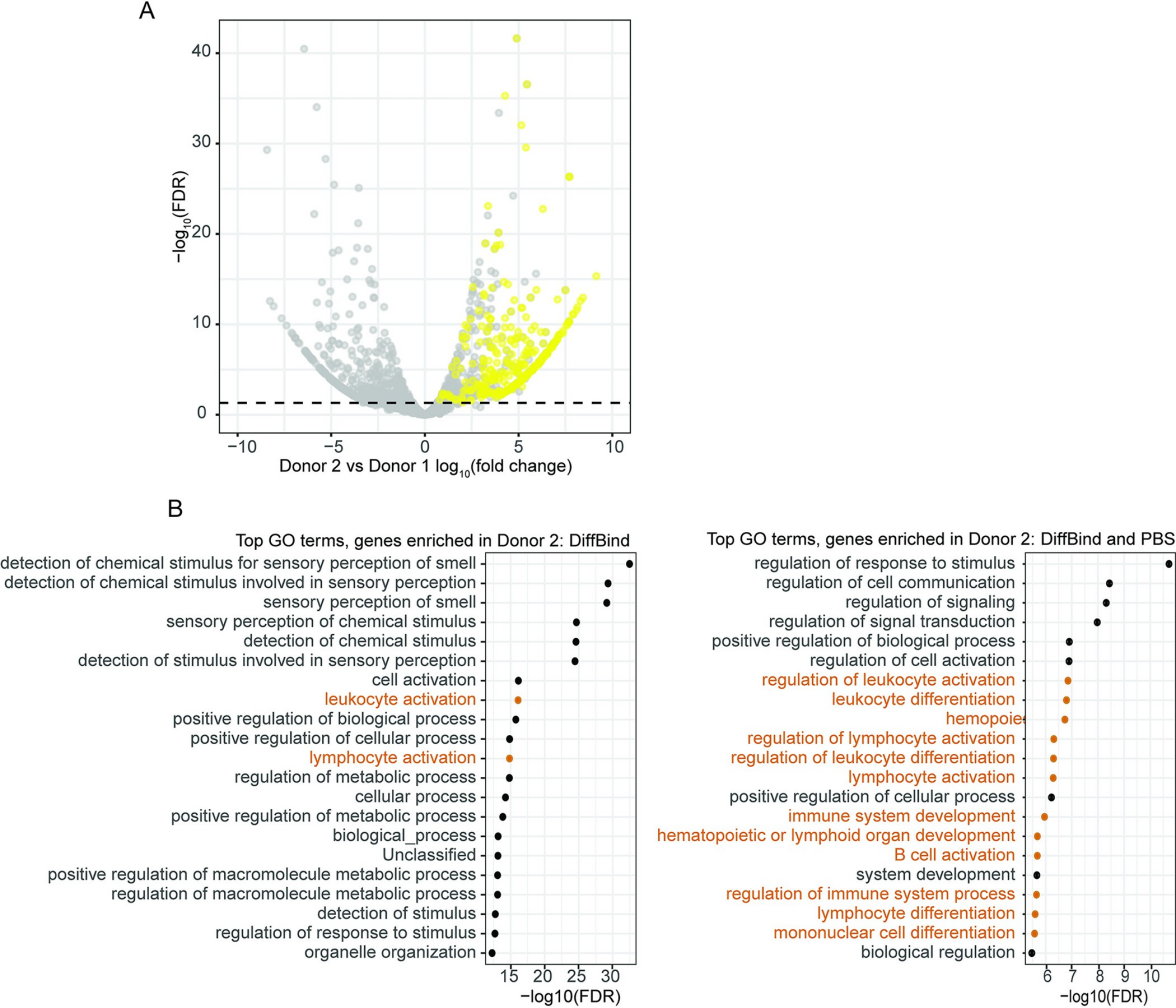
Combining DiffBind with a PBS-based differential analysis. (A) Volcano plot showing significantly differentially bound regions in tissue samples from two ENTEx donors (gray). Yellow highlighted points indicate regions enriched in donor 2 that also overlap with bins that have a difference in PBS > 0.9. Values have been randomly downsampled to 10000 points to facilitate visualizing trends. (B) Comparison between top GO terms for a DiffBind-based differential analysis (left) and a combined DiffBind and PBS-based analysis (right). Terms explicitly relating to immune system processes are highlighted in orange. The FDR-adjusted p values in the right panel are greater (less significant) than those in the left panel due to the smaller number of input genes.

We compared small intestine H3K27ac ChIP-seq from two different ENTEx donors (JKYN and LVAN, here referred to as donor 1 and donor 2) [19]. Though samples from the same tissue from different donors are often assumed to be similar enough to be treated as biological replicates, differences in the cell type composition of the samples, donor genetics, or donor epigenetics could all undermine the validity of this assumption. We first called peaks in samples from each donor, then used DiffBind with DESeq2 to identify differentially bound regions (Fig 5A), and finally looked for overlap between these differential regions with nearby genes. We identified ~38,000 differentially bound regions with DiffBind (FDR < 0.05), corresponding to ~11,000 genes.

We then calculated the difference in PBS values between donor samples, and intersected the differentially bound regions identified with DiffBind with the differential PBS bins (Fig 5A). We considered a difference in PBS of greater than 0.9 or less than -0.9 to be a meaningful difference in signal. In general, we observed that higher fold changes and more significant FDR values from DiffBind corresponded to larger differences in PBS. However, only a minority of the regions that were identified as significantly differentially enriched with DiffBind overlapped with bins with a difference in PBS with a magnitude greater than 0.9, approximately 9000 in total, corresponding to ~3000 genes.

Both the standalone DiffBind analysis and the combined PBS-DiffBind analysis suggested a considerable difference in composition between the samples from the two donors, and we therefore sought to identify potential patterns relating to biological processes shared among the differential regions. We ran a GO-enrichment analysis for the lists of genes intersecting differentially bound regions from the DiffBind analysis or the DiffBind with PBS analysis (Fig 5B). We found ~425 significantly enriched GO terms when using all regions identified by DiffBind, with no clear trends regarding biological process or organ system. However, when we used the intersection of PBS with DiffBind, we found that the terms (~245) were more limited to those relating to immune processes in donor 2, with no clear trends in donor 1 (S1 and S2 Tables). These data suggested that immune cells comprise a larger fraction of the donor 2 small intestine sample compared to the donor 1 sample.

### Understanding tissue composition by combining PBS with GWAS SNPs and phenotypes

We next sought an orthogonal approach for evaluating whether the differences in H3K27ac profiles identified via the DiffBind-PBS analysis result from a difference in tissue composition between the two donor samples. Our approach involved combining regulatory element information, represented by H3K27ac PBS values, and genetic variants associated with diverse phenotypes, using stratified LD-score regression (s-LDSC) [29, 30]. Genetic variants associated with a phenotype are often found in regulatory elements that are active in a cell or tissue type connected with that phenotype [29]. Using s-LDSC, we can quantitatively evaluate these relationships. Typically, s-LDSC analyses are used to better understand phenotype-tissue relationships using a set of well-characterized tissue-specific annotations [29]. Here, we use s-LDSC to better understand a tissue-specific annotation using already established tissue-phenotype relationships. For example, we expect to see a strong association between H3K27ac annotations from white blood cells and a white-blood cell-counts phenotype.

**Fig 6:**
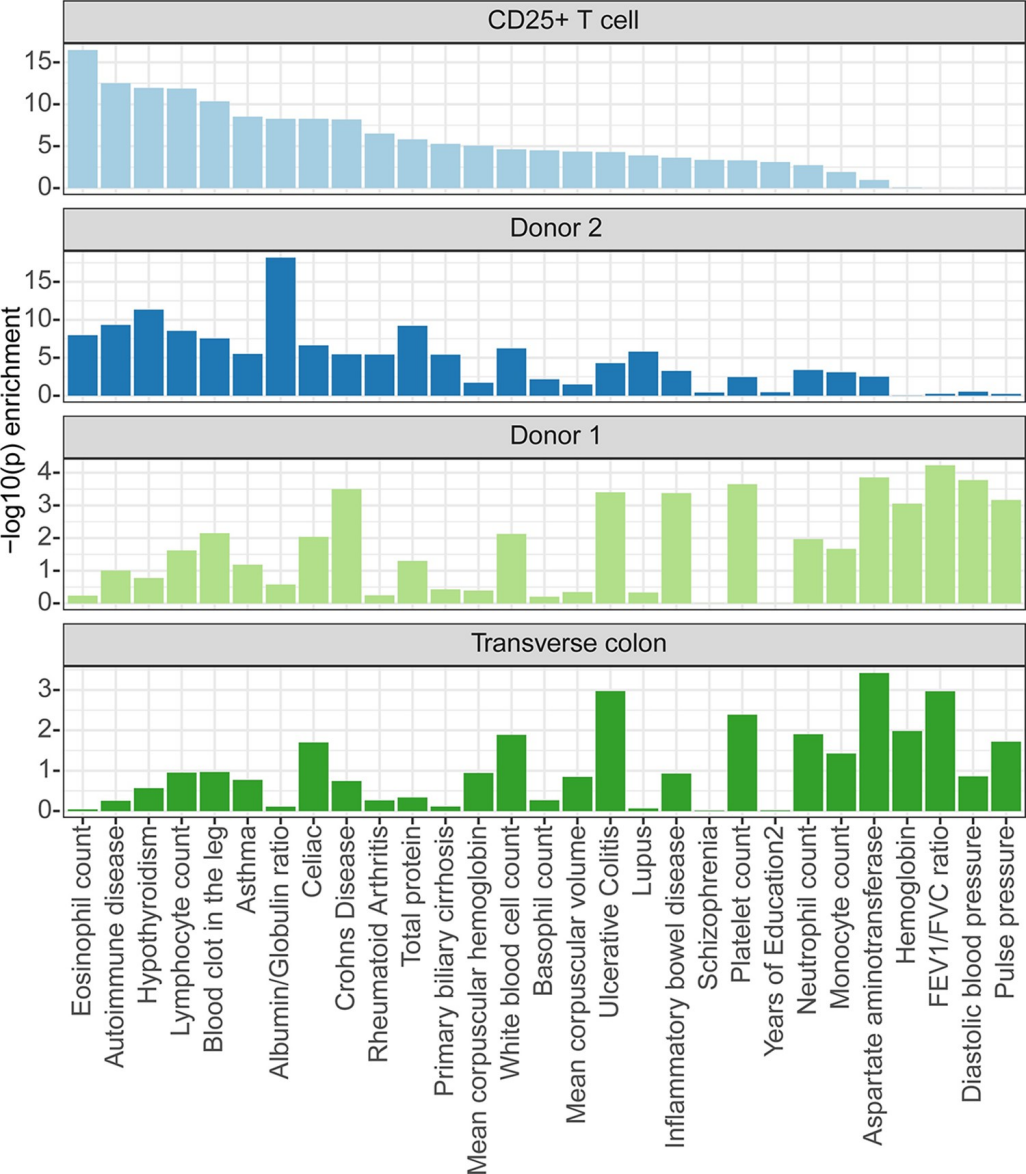
S-LDSC using PBS-based annotations. S-LDSC results describing phenotypic enrichments of H3K27ac annotations for a representative immune cell type (CD25+ T cell), two small intestine samples from two different ENTEx donors, and a representative gastrointestinal sample (transverse colon, or colon TV). The enrichment pattern for phenotypes in donor 2 H3K27ac more closely matches that of the CD25+ T cells, whereas the enrichment pattern for donor 1 more closely matches that of transverse colon.

We ran s-LDSC with the donor 1 and donor 2 small intestine H3K27ac datasets and a collection of phenotypes from publicly available summary statistics [19] (Fig 6). We found that the PBS annotations (thresholded with PBS > 0.9) were easier to include in our pipeline than peak calls, since they required no adjustment for window size or read depth. The small intestines annotations from both donor 1 and donor 2 showed similar levels of enrichments for variants associated with Crohn’s disease, ulcerative colitis, and inflammatory bowel disease, all related phenotypes resulting from intestinal inflammation. Only donor 2, however, showed an even stronger enrichment for immune phenotypes. We compared the enrichment patterns in donor 1 and donor 2 with representative tissues relating to either the gastrointestinal or immune systems, and found that donor 2 most resembled an inflammatory immune cell type (CD25+ T cells) while donor 1 resembled a gastrointestinal tissue type (transverse colon) (Fig 6 see S6 Fig for a comparison with a larger set of cell types and tissues). This confirmed our suspicion of the difference in tissue composition between donor 1 and donor 2, with a considerable fraction of the donor 2 sample consisting of inflammatory cell types.

We then consulted the clinical pathology notes for the different donor tissue samples [28], and observed that the donor 2 sample had half a dozen lymphoid nodules noted, whereas donor 1 had only scattered lymphoid aggregates, suggesting a considerably higher fraction of lymphoid tissue in donor 2. Interestingly, though the small intestine samples are categorized as small intestine on GTEX, in ENCODE/ENTEx the tissues are categorized as Peyer’s Patch [19], which is a region of the small intestine that is rich in lymphoid tissue. Our analysis is consistent with the well-described notion of bulk tissue sample heterogeneity, which is often used to demonstrate the importance of single-cell methods. However, single cell methods are currently more costly and experimentally challenging than bulk, and are not always feasible to conduct in certain tissue types. Moreover, a considerable amount of bulk tissue data has already been generated, and can still be mined for valuable biological insights. We show here that by comparing multiple bulk tissue samples with orthogonal analysis methods, albeit ones that are designed exclusively for analyzing bulk tissue, we can better understand their composition even in the absence of single cell data.

## Discussion

Here, we present a straightforward method to analyze bulk ChIP-seq data for a range of histone modifications. Our bin-based method is lower resolution compared to peak calling, but is versatile, interpretable, and fast. Moreover, our method is particularly advantageous with very broad, low signal, as can be seen with H3K27me3 silencing in differentiated cell types, which is often missed by the most commonly used peak callers. An analysis method which can be used for both narrow and broad histone marks can considerably simplify computational pipelines; we have also found that PBS can be used effectively with ATAC-seq without any additional modifications (S3 Fig) [31]. We have found our method to be complementary to peak calling for narrow marks, but a potential substitute for peak calling for broader marks such as H3K27me3.

A sample analysis can begin with using PBS to visualize a large number of datasets over a large region in a format similar to a heatmap. PBS-based visualizations are not only efficient and compact, but they also avoid many of the potential pitfalls associated with normalizing data, since the values range from zero to one for all tracks. With simple arithmetic, both local and global between-track differences can be identified within moments, which can be useful prior to designing a more time- and computationally-intensive DESeq2 or edgeR-based differential analysis.

In this work, we have not experimented with different bin sizes, largely due to our having met our objective of 5 kB being a bin size that can be used to detect signal in a range of broad and narrow marks. For relatively modest changes (bin sizes of 3kb, 5kb, or 10kb), we expect the value of PBS itself at a particular location in the genome not to change dramatically, though we anticipate that significantly larger bins sizes may cause some regions of enrichment to get diluted and cause PBS to decrease. Conversely, bins that are too small may lead to a different problem: the number of reads in any given bin can be very low, and the discrete nature of the distribution of read counts becomes important (S7 Fig). Therefore, the continuous-valued gamma distribution would no longer be an appropriate approximation of the data, and the PBS method should be modified to accommodate a different family of distributions.

Recent work has highlighted the tendency of commonly-used differential expression methods to report false positives; often the most effective means of using these methods is selecting the top several dozen most significant sites and ignoring the remainder [27]. However, using PBS to narrow the number of candidate sites provides a more principled and mechanistic interpretation: whereas many current methods test for differences in peak magnitudes, differences in PBS indicate transition between presence and absence of signal (Fig 5). For example, regions highlighted by DiffBind that have a difference in PBS of 0.9 or more will not include those that have a difference in peak magnitudes, even if both samples have peaks. The resulting list will be limited to regions that have a peak in one sample, indicating the presence of an active regulatory element, and no signal in the second.

Furthermore, we extend our applications of PBS to interrogate and characterize GWAS variants of known phenotypes. The vast majority of variants identified via GWAS land in non-coding regions, which underscores the potential for insights into both the phenotypes and the non-coding regions themselves by integrating ChIP-seq data with SNPs identified from GWAS [32]. Whereas s-LDSC is often used to better understand phenotypes based on tissue-specific annotations, here we demonstrate that s-LDSC with well-characterized phenotypes can also be used to better understand tissue composition. Although s-LDSC must be used alongside GWAS that are sufficiently well-powered (n > 10000), we have found that sufficient datasets of this size or larger are publicly available to enable our analysis.

We developed PBS to support the data production effort of the Broad Institute’s Epigenomics production group, which produces a large number of high-quality ChIP-Seq datasets each year, and we have found it very valuable for downstream data evaluation and analysis. We have described only a small number of a range of possible applications of the PBS method here. We anticipate that its simplicity, speed, and versatility will prove useful to many seeking to analyze ChIP-seq and related data types.

## Methods

### Binning

We binned aligned ChIP-seq reads (BAM file format) into non-overlapping 5 kB intervals using the count function in Integrative Genomics Viewer tools (version 2.8.2, http://broadinstitute.org/igvtools), with low-mappability reads excluded (MAPQ < 1). We then converted the output from IGV tools count from a bigwig to a bed file format using the UCSC Genome Browser executable bigwigAverageOverBed (http://hgdownload.soe.ucsc.edu/admin/exe/linux.x86_64/), and excluded bins overlapping with blacklisted regions defined by ENCODE (https://sites.google.com/site/anshulkundaje/projects/blacklists).

### Mappability rescaling

We next rescaled the read-counts in each bin by the average mappability of the bin, which describes how uniquely reads can align to the genomic sequence overlapping the bin. This step is one of two, the second being CNV rescaling, in which we incorporate local information to improve our global estimate. For a read to be mapped uniquely to a position in the genome, the genomic sequence starting at that position and extending for the length of the read needs to be unique within the reference genome assembly. The per-bin mappability is the fraction of base positions within the bin where reads can be uniquely aligned. It increases with sequence uniqueness and read length, and is higher for paired-end than for single-end data. Low MAPQ (multimapped) reads will align to low mappability bins; because we filter low MAPQ reads when calculating read-counts, low mappability bins will have a read-counts deficit. To correct for the effect of mappability on read-counts, we calculated a scaling factor, or mappability score, based on publicly available estimates of per-base pair mappability for commonly used read lengths (Genome Multitool for read lengths 36 bp or 75 bp, and Umap for read lengths of 25, 50 or 100 bp) [33, 34]. Paired-end reads do not have well-defined read-lengths, and so we used mappability values for 100 bp reads for all paired-end data. Mappability scores ranged from 0 (not mappable) to 1 (uniquely mappable). Bins with mappability scores less than 0.5 are excluded, and counts in bins with mappability scores between 0.5 and 1 are rescaled by dividing by the mappability score.

### CNV rescaling

Following mappability rescaling, we rescaled per-bin read-counts for copy number variations (CNVs). CNVs, most often resulting from genomic instability characteristic of tumor samples or cancer cell lines, can lead to increases or decreases in read-counts which can be confused with signal enrichment (or depletion). We ran CNV detection and ploidy estimation steps on input control tracks, to avoid confusion between CNVs and true enrichment; if CNVs were detected, we used the ratios resulting from the ploidy estimation step to correct the epitope tracks for matching biosamples.

We detected CNVs in a sample by grouping the 5 kB bins into 20 subsets, and then used Hartigans’ dip test to determine multimodality of the distribution of read-counts in any of the subsets [35]. By using subsets that do not correspond to chromosome size, we are able to detect of CNVs resulting from the loss of entire chromosomes, as well as those in small regions (~10s of kB). This CNV detection step is very rapid (< 1 second), and if no CNVs are detected, the computationally intensive ploidy estimation step is skipped. To estimate ploidy for datasets with CNVs, we used the CNAnorm package [36]. Briefly, CNAnorm fits a multimodal normal distribution to a distribution of read-counts, defines diploid as the mode with the highest frequency of read-counts, and outputs a value for each bin indicating its fold-change from diploid. These fold-change values are saved as a bed file, and used to rescale the input control file, as well as any epitope tracks using the biosample of the input control.

### Gamma distribution fitting to background

We characterized the background read-counts distribution by fitting a gamma distribution to the bins with a read-count value less than or equal to a threshold θ, which we set to be the median of the data. We assumed that read-counts less than θ represent only background, but also found that enough of the shape of the gamma distribution would be preserved with this threshold to allow us to estimate its parameters (shape (α) and rate (β)). This step of the pipeline was implemented by minimizing a modified Cramer-von-Mises criterion ω^2^, which we defined as the area of difference between two cumulative distribution functions,

$$\omega^{2}=\int_{-\infty }^{\theta }{\left[F(x)-\lambda F^{*}(x;\alpha ,\beta )\right]}^{2}dF^{*}(x;\alpha ,\beta )$$

where F(x) represents the empirical cumulative distribution function (CDF) of the observed data, F*(x; α, β) represents the estimated CDF with parameters a and b, and λ represents the fraction of data corresponding to background. Using a non-linear optimizer (optim) in R, we estimated values for α, β and λ.

### PBS calculation

Using the parameters calculated in the previous step, we calculated a per-bin value of PBS using the following equation,

$${PBS}_{x}=\frac{f(x)-\lambda f^{*}(x;\alpha ,\beta )}{f(x)},if\;f(x)>\lambda f^{*}(x;\;\alpha ,\beta );0\;otherwise$$

where f(x) represents the empirical probability density function (PDF), f*(x; α, β) represents the PDF of the estimated background distribution, and λ represents the genome-wide fraction of the bins corresponding to background (Fig 1A).

### Fit quality estimation

To identify cases in which the PBS fit fails, we provide a fit quality metric based on the area for which the estimated distribution f*(x) exceeds that of the empirical distribution f(x) (S8 Fig). This is in contrast to the PBS measurement, which defines signal based on the amount that f(x) exceeds f*(x). The fit quality metric is calculated with the equation shown below,

$$q=\int_{for\;x\;s.t.x>\theta \;and\;f^{*}>f}^{}f^{*}(x;\alpha ,\beta )-\lambda f(x)dx$$

The fit metric is calculated after the optimization step used to estimate the background gamma distribution parameters. Additionally, while the background is optimized to fit to the bottom fiftieth percentile of the data, the fit metric is calculated based on the top fiftieth percentile. In our experience, a good fit is defined by a residual area of less than 0.05. Some examples of high, medium, and low-quality fits are shown in S8 Fig.

### Additional bioinformatics analyses

Peak calling for narrow histone marks was conducted using MACS2 default parameters (version 2.2, https://github.com/macs3-project/MACS). Peak calling for broad histone marks was conducted using MACS2 default parameters and the “—broad” flag. DiffBind analysis was run with default parameters. LDSC was run according to instructions described in https://github.com/bulik/ldsc.

## Supporting information

S1 Fig Rescaling corrects for the effects of low mappability and copy number variation. (A) Low mappability bins tend to be enriched in low values of read-counts (orange box) due to an underestimate of reads caused by uncounted multi-mapped reads. Rescaling these bins based on the mappability scores of the underlying sequence corrects for this bias. (B) Copy number variations create additional modes in an input control sample originating from tumor tissue, which disappear once bins overlapping with CNVs are rescaled by estimated ploidy of each bin.
(TIF)

S2 Fig Example gamma distribution fit to an input control dataset. The gamma distribution representing background encompasses the input control dataset in its entirety.
(TIF)

S3 Fig Calculating PBS for an ATAC-seq dataset. (A) Example distributions of bin read-counts and PBS for three ATAC-seq datasets from [31]. (B) Overlay of reads and PBS at an example immune-related locus, demonstrating how PBS accurately represents the ATAC-seq signal.
(TIF)

S4 Fig Comparing PBS and peak calling for an H3K27ac dataset. (A) Histogram of per-bin read-counts annotated based on whether a bin overlaps with a peak. The inset shows the detail of the right-hand tail of the distribution. Bins with higher read-counts usually fall outside the estimated background distribution (solid black line) and overlap with peaks. (B) Histogram of per-bin PBS for the same dataset. Most bins overlapping with peaks have a PBS = 1, suggesting that most bins with peaks have signal in this dataset.
(TIF)

S5 Fig Multi-epitope, multi-dataset comparison between peaks and PBS. Forty datasets from a range of different cell types and 4 histone marks are shown. (A) Distribution of bins overlapping with peaks in each of three categories of PBS (high, PBS > 0.9; moderate, 0.9 ≥ PBS > 0.1; and low, 0.1 ≥ PBS), demonstrating that almost all bins with peaks have high PBS. The median number of peak-overlapping bins genome-wide with low PBS in each histone mark is as follows: 248 in H3K27ac; 145 in H3K27me3; 316 in H3K36me3; and 357 in H3K4me1. The fraction of bins with low PBS appears higher in H3K27me3 relative to the other histone marks due to the small number of total bins genome-wide with called peaks. (B) Distribution of bins not overlapping with peaks and three categories of PBS. The majority of bins not overlapping with peaks have low PBS (< 0.1). A consistent fraction of peaks in H3K27ac, H3K36me3, and H3K4me1 overlap with bins with moderate PBS and high PBS. The distribution of overlap of peak-free bins with regions of moderate and high PBS is more variable across samples for H3K27me3, with some datasets showing close to 50% overlap between peak-free bins and moderate PBS.
(TIF)

S6 Fig Relationship between s-LDSC phenotypic enrichments in Donor 1 and Donor 2 small intestine and a set of immune and gastrointestinal tissues and cell types. Values in the heatmap represent correlations between -log_10_(p) of enrichments in phenotypes shown in Fig 6 in each cell or tissue type shown in the fig. Small intestine from Donor 2 clusters with immune-related cell types and small intestine from Donor 1 cluster with GI-related cell types.
(TIF)

S7 Fig Small bin sizes result in a discrete distribution. Using small bin sizes leads to a discretized distribution which can no longer be estimated with a continuous gamma distribution. The peaks at every 0.25 read-counts represent increments of a single 75 bp read in a 300 bp bin. The dataset shown is the same as shown in Fig 2C in the main manuscript.
(TIF)

S8 Fig Estimating the fit quality of the estimated gamma distribution using the fit quality metric. Examples of high, medium and low-quality fits, as quantified by the residual area metric, of an estimated gamma distribution (solid black line) to the background distribution of bin read counts. The dashed black line represents the median read-counts of the dataset.
(TIF)

S1 Table GO enrichment analysis for genes overlapping bins identified as differentially enriched via DiffBind (adjusted p value < 0.05). (TXT)

S2 Table GO enrichment analysis for genes overlapping bins identified as differentially enriched via DiffBind (adjusted p value < 0.05) and overlapping a bin with a difference in PBS of > 0.9. (TXT)
