# Supplementary figures and images for: Analyzing histone ChIP-seq data with a bin-based probability of being signal
Source: PLoS Comput Biol. 2023 Oct 20;19(10):e1011568. doi: 10.1371/journal.pcbi.1011568 (PMC10619820; doi:10.1371/journal.pcbi.1011568)

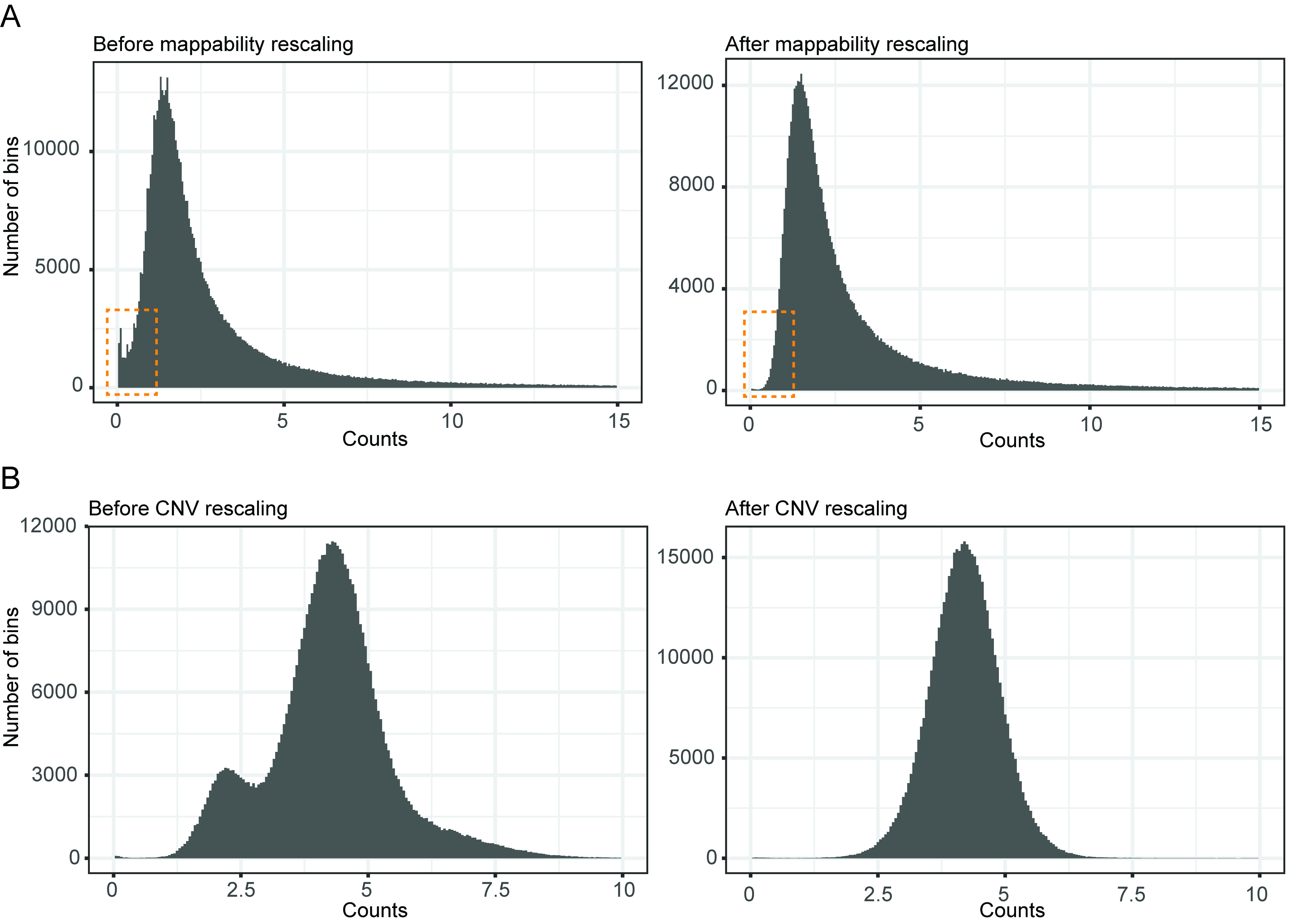

Supplement: S1 Fig — (A) Low mappability bins tend to be enriched in low values of read-counts (orange box) due to an underestimate of reads caused by uncounted multi-mapped reads. Rescaling these bins based on the mappability scores of the underlying sequence corrects for this bias. (B) Copy number variations create additional modes in an input control sample originating from tumor tissue, which disappear once bins overlapping with CNVs are rescaled by estimated ploidy of each bin. (TIF) [file pcbi.1011568.s001.tif]

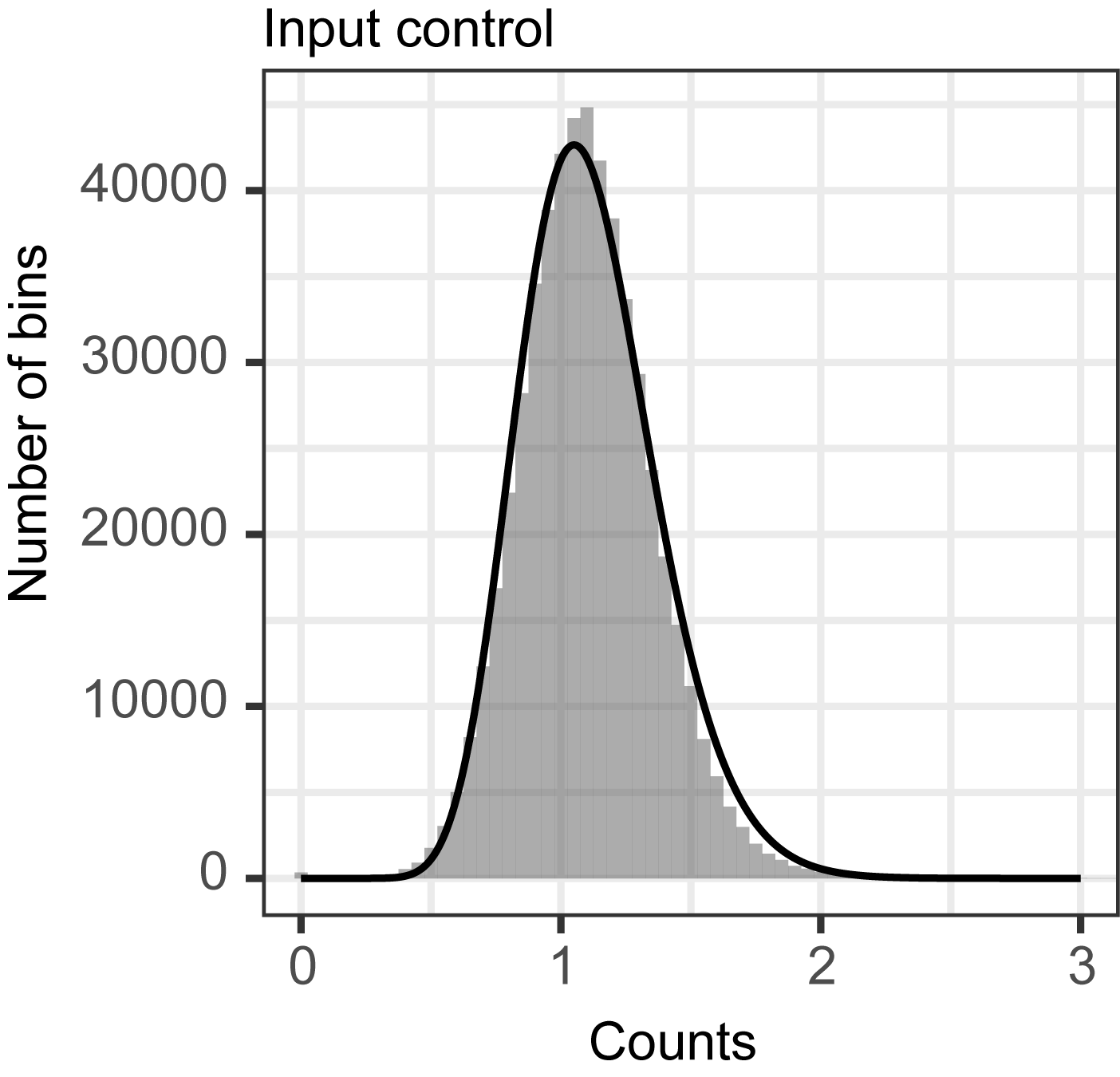

Supplement: S2 Fig — The gamma distribution representing background encompasses the input control dataset in its entirety. (TIF) [file pcbi.1011568.s002.tif]

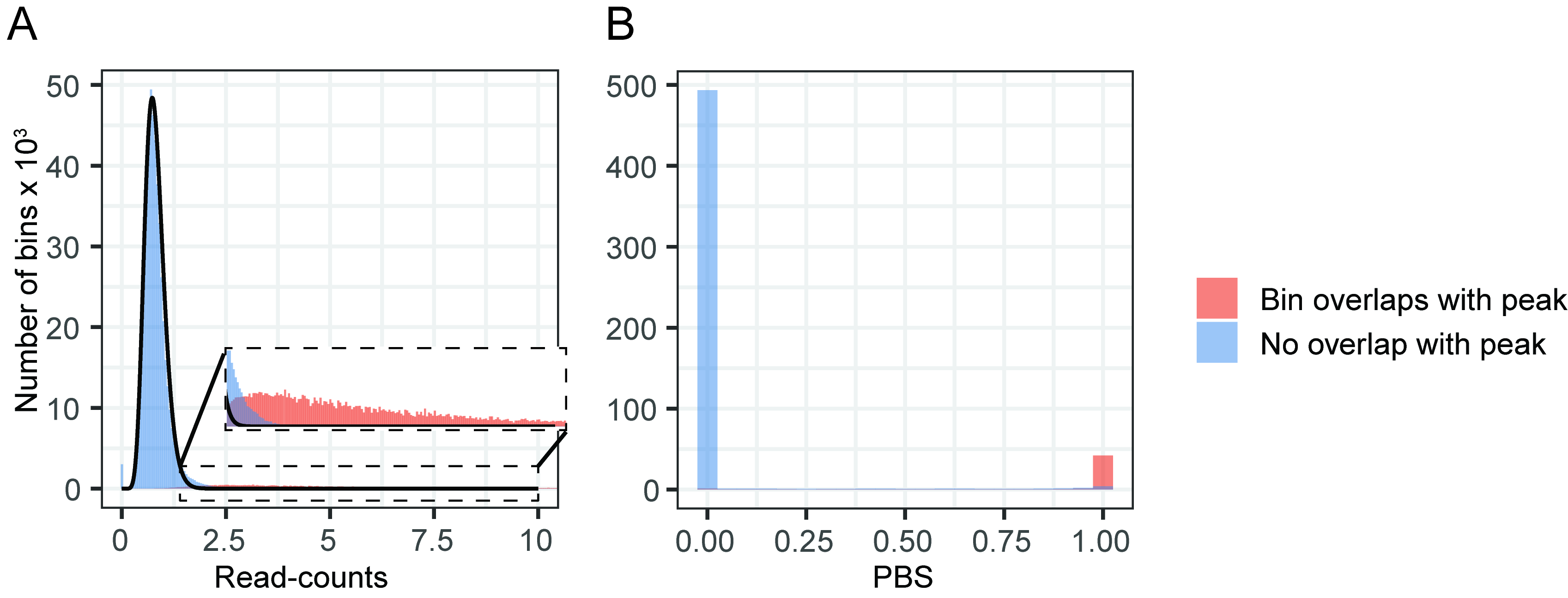

Supplement: S4 Fig — (A) Histogram of per-bin read-counts annotated based on whether a bin overlaps with a peak. The inset shows the detail of the right-hand tail of the distribution. Bins with higher read-counts usually fall outside the estimated background distribution (solid black line) and overlap with peaks. (B) Histogram of per-bin PBS for the same dataset. Most bins overlapping with peaks have a PBS = 1, suggesting that most bins with peaks have signal in this dataset. (TIF) [file pcbi.1011568.s004.tif]

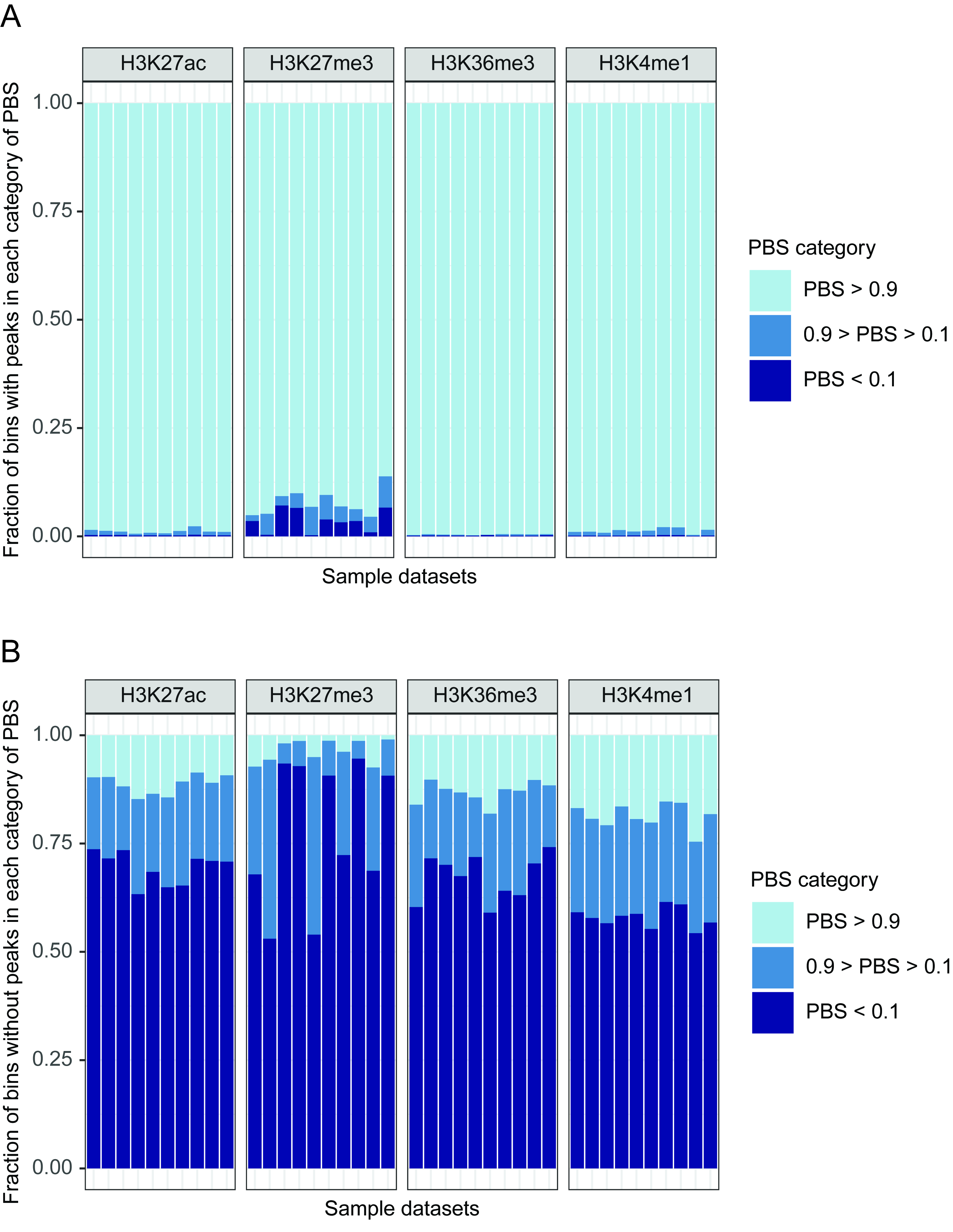

Supplement: S5 Fig — Forty datasets from a range of different cell types and 4 histone marks are shown. (A) Distribution of bins overlapping with peaks in each of three categories of PBS (high, PBS > 0.9; moderate, 0.9 ≥ PBS > 0.1; and low, 0.1 ≥ PBS), demonstrating that almost all bins with peaks have high PBS. The median number of peak-overlapping bins genome-wide with low PBS in each histone mark is as follows: 248 in H3K27ac; 145 in H3K27me3; 316 in H3K36me3; and 357 in H3K4me1. The fraction of bins with low PBS appears higher in H3K27me3 relative to the other histone marks due to the small number of total bins genome-wide with called peaks. (B) Distribution of bins not overlapping with peaks and three categories of PBS. The majority of bins not overlapping with peaks have low PBS (< 0.1). A consistent fraction of peaks in H3K27ac, H3K36me3, and H3K4me1 overlap with bins with moderate PBS and high PBS. The distribution of overlap of peak-free bins with regions of moderate and high PBS is more variable across samples for H3K27me3, with some datasets showing close to 50% overlap between peak-free bins and moderate PBS. (TIF) [file pcbi.1011568.s005.tif]

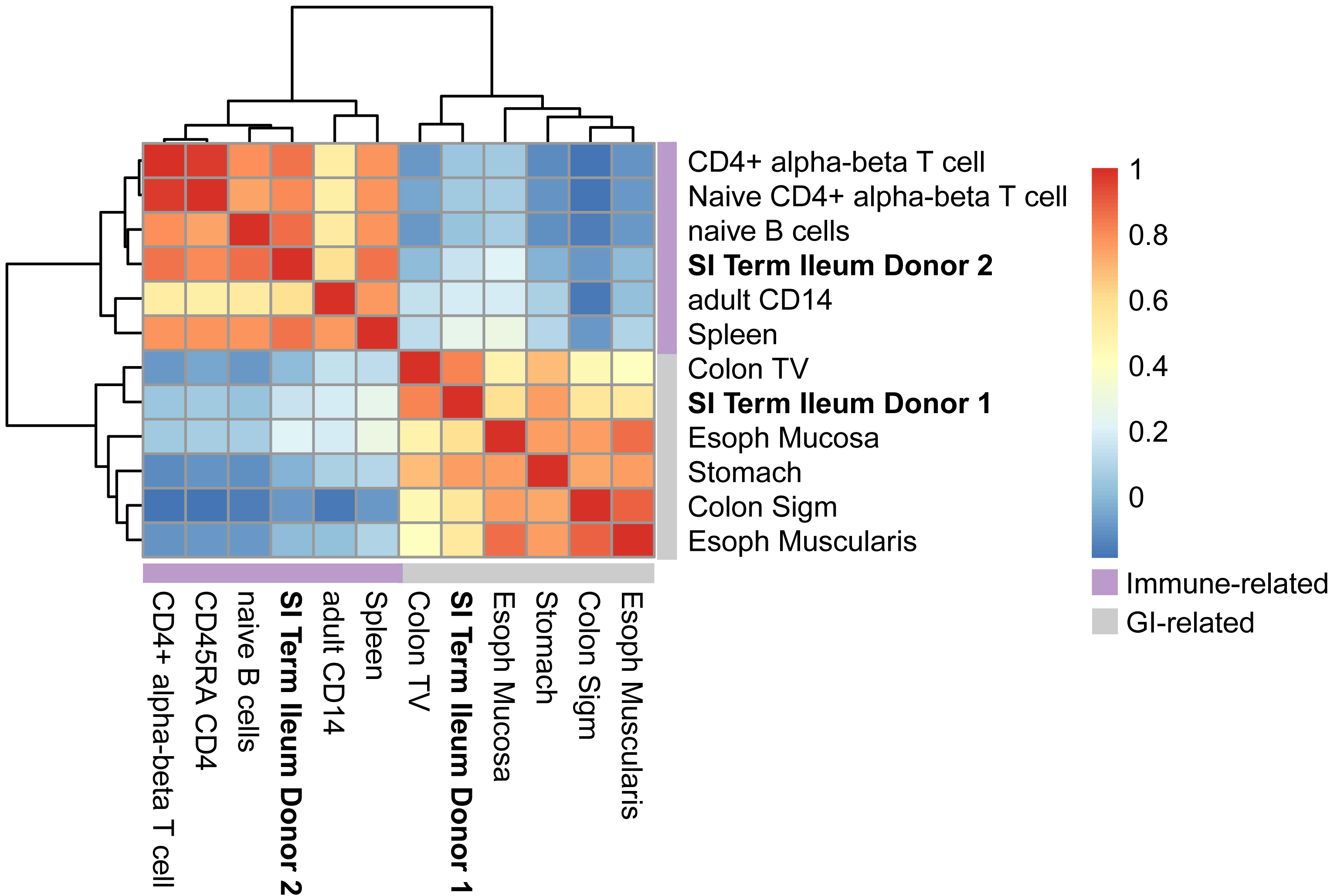

Supplement: S6 Fig — Values in the heatmap represent correlations between -log10(p) of enrichments in phenotypes shown in Fig 6 in each cell or tissue type shown in the fig. Small intestine from Donor 2 clusters with immune-related cell types and small intestine from Donor 1 cluster with GI-related cell types. (TIF) [file pcbi.1011568.s006.tif]

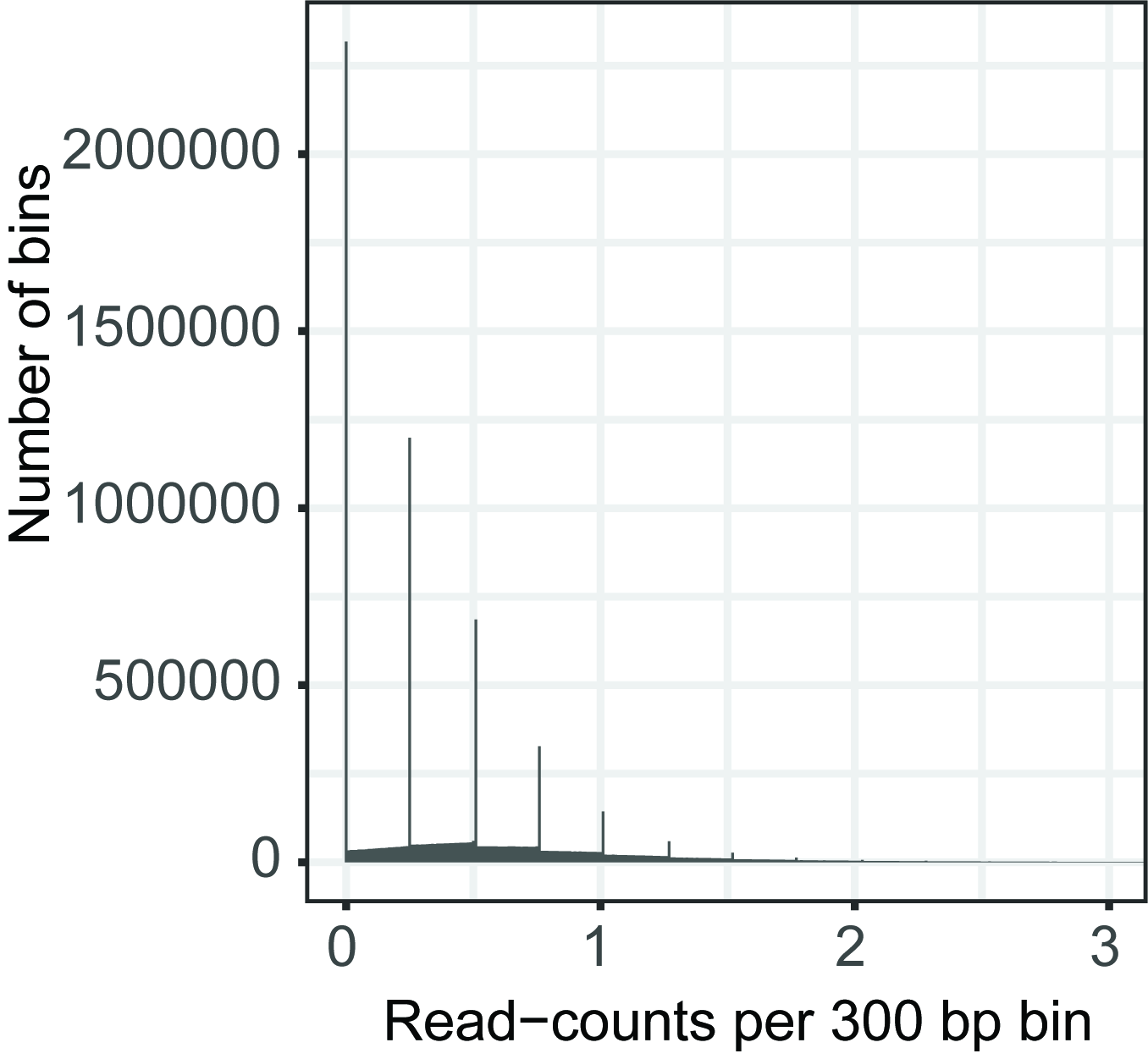

Supplement: S7 Fig — Using small bin sizes leads to a discretized distribution which can no longer be estimated with a continuous gamma distribution. The peaks at every 0.25 read-counts represent increments of a single 75 bp read in a 300 bp bin. The dataset shown is the same as shown in Fig 2C in the main manuscript. (TIF) [file pcbi.1011568.s007.tif]

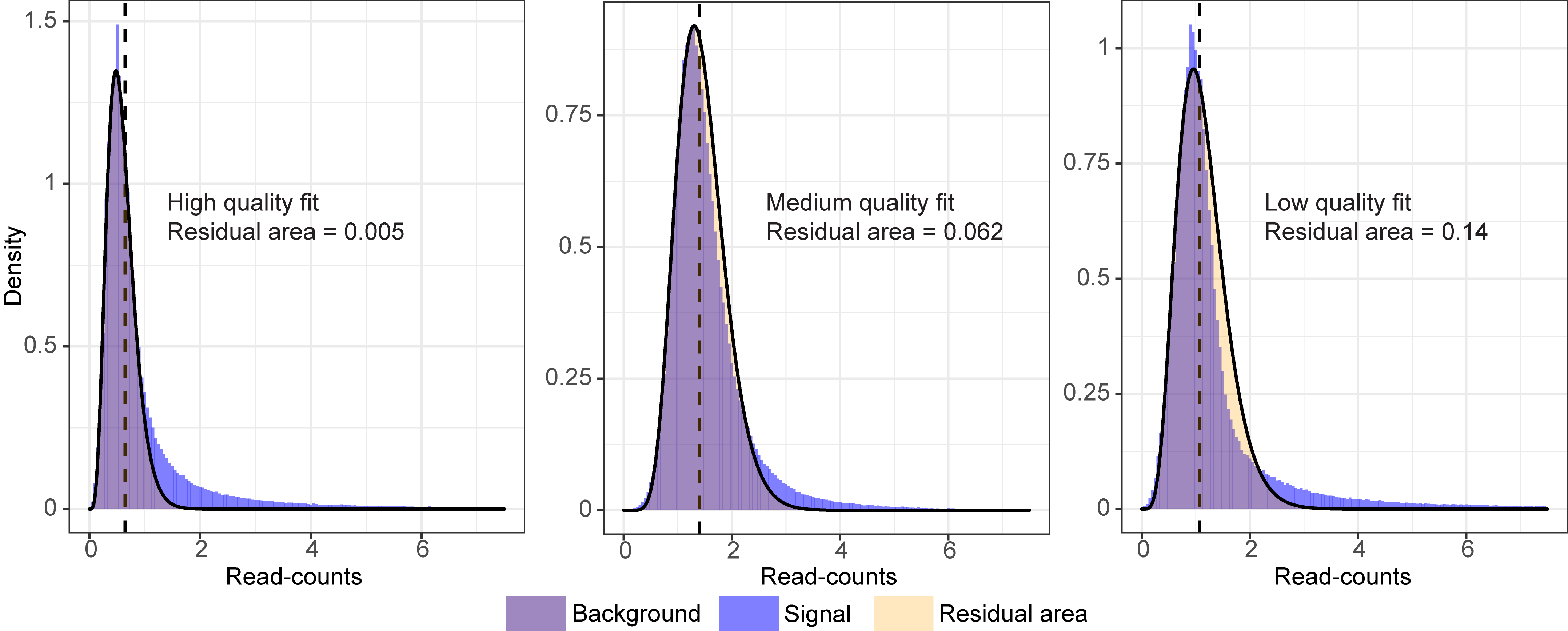

Supplement: S8 Fig — Examples of high, medium and low-quality fits, as quantified by the residual area metric, of an estimated gamma distribution (solid black line) to the background distribution of bin read counts. The dashed black line represents the median read-counts of the dataset. (TIF) [file pcbi.1011568.s008.tif]
